# Amorphous cellulose nanofiber supercapacitors

**DOI:** 10.1038/s41598-021-85901-3

**Published:** 2021-03-19

**Authors:** Mikio Fukuhara, Tomoyuki Kuroda, Fumihiko Hasegawa, Toshiyuki Hashida, Mitsuhiro Takeda, Nobuhisa Fujima, Masahiro Morita, Takeshi Nakatani

**Affiliations:** 1 New Industry Creation Hatchery Center, Tohoku University, Sendai, 980-8579 Japan; 2 Fracture and Reliability Research Institute, Graduate School of Engineering, Tohoku University, Sendai, 980-8579 Japan; 3 National Institute of Technology, Sendai College, Natori, 981-1239 Japan; 4 Faculty of Engineering, Shizuoka University, Hamamatsu, 432-8561 Japan; 5 Cellulose Nanofiber Research Laboratory, Nippon Paper Industries, Fuji, 417-8520 Japan

**Keywords:** Biophysics, Microbiology, Structural biology

## Abstract

Despite the intense interest in cellulose nanofibers (CNFs) for biomedical and engineering applications, no research findings about the electrical energy storage of CNF have been reported yet. Here, we present the first electroadsorption effects of an amorphous cellulose nanofiber (ACF) supercapacitor, which can store a large amount of electricity (221 mJm^−2^, 13.1 Wkg^−1^). The electric storage can be attributed to the entirely enhanced electroadsorption owing to a quantum-size effect by convexity of 17.9 nm, an offset effect caused by positive polar C_6_=O_6_ radicles, and an electrostatic effect by appearance of the localised electrons near the Na ions. The supercapacitor also captures both positive and negative electricity from the atmosphere and in vacuum. The supercapacitor could illuminate a red LED for 1 s after charging it with 2 mA at 10 V. Further gains might be attained by integrating CNF specimens with a nano-electromechanical system (NEMS).

## Introduction

Recently, the examination of material and biomedical properties of biodegradable cellulose nanofibers (CNFs) that are thermally stable, strong durable, and light-weight has gained significant attention^1–4^. The completely individualised CNFs were developed by 2,2,6,6-tetramethylpiperidine-1-oxyl radical (TEMPO)-mediated oxidation under aqueous conditions^5^. Although CNFs are assumed to be renewable with innumerable promising applications^6^, no research findings about the electrical energy storage of CNF have been reported yet, except for the use of cellulose separators in lithium-ion batteries (LIBs)^7,8^. Recently, we found that amorphous titanium-oxides (golden, a-TiO_2-x_, ATO)^9,10^, perfluorinated polymer (APP)^11^, and aluminium-oxides (blackish, a–Al_2-y_O_3-z_, AAO) supercapacitors^12–15^, showed one hundred times higher capacitance than that of a conventional aluminium electrolytic capacitor^16^, illumination of red LED light^11^, and a capturing effect of a positive and negative charges from the atmosphere^﻿12﻿^, respectively. Especially, the AAO is suitable for applications of heavy electricity such as stationary storage batteries. These amorphous capacitors are completely different from conventional “wet” cells, such as electric double-layer capacitors (EDLCs) and LIBs, which are controlled through the diffusivity of ions^14,15^. However, they are artificial products which are not environmentally friendly. If we can provide a supercapacitor composed of renewable papers, which are made by dewatering a dilute suspension of cellulose fibres, it could cast new light on paper electronics. Strictly speaking, however, there is no data about electroadsorption of paper, especially CNF. Herein, we report the high-performance electric storage of “dry” amorphous cellulose nanofibers (ACF). Our results show that the insulating ACF specimen with nanometre-sized cavities and high work function, is an ideal candidate for supercapacitors with potential applications of light electricity such as handheld electronic devices, transportation, and renewable energy storage for power grids.

## Results and discussion

### An electric storage system of ACF

**Figure 1:**
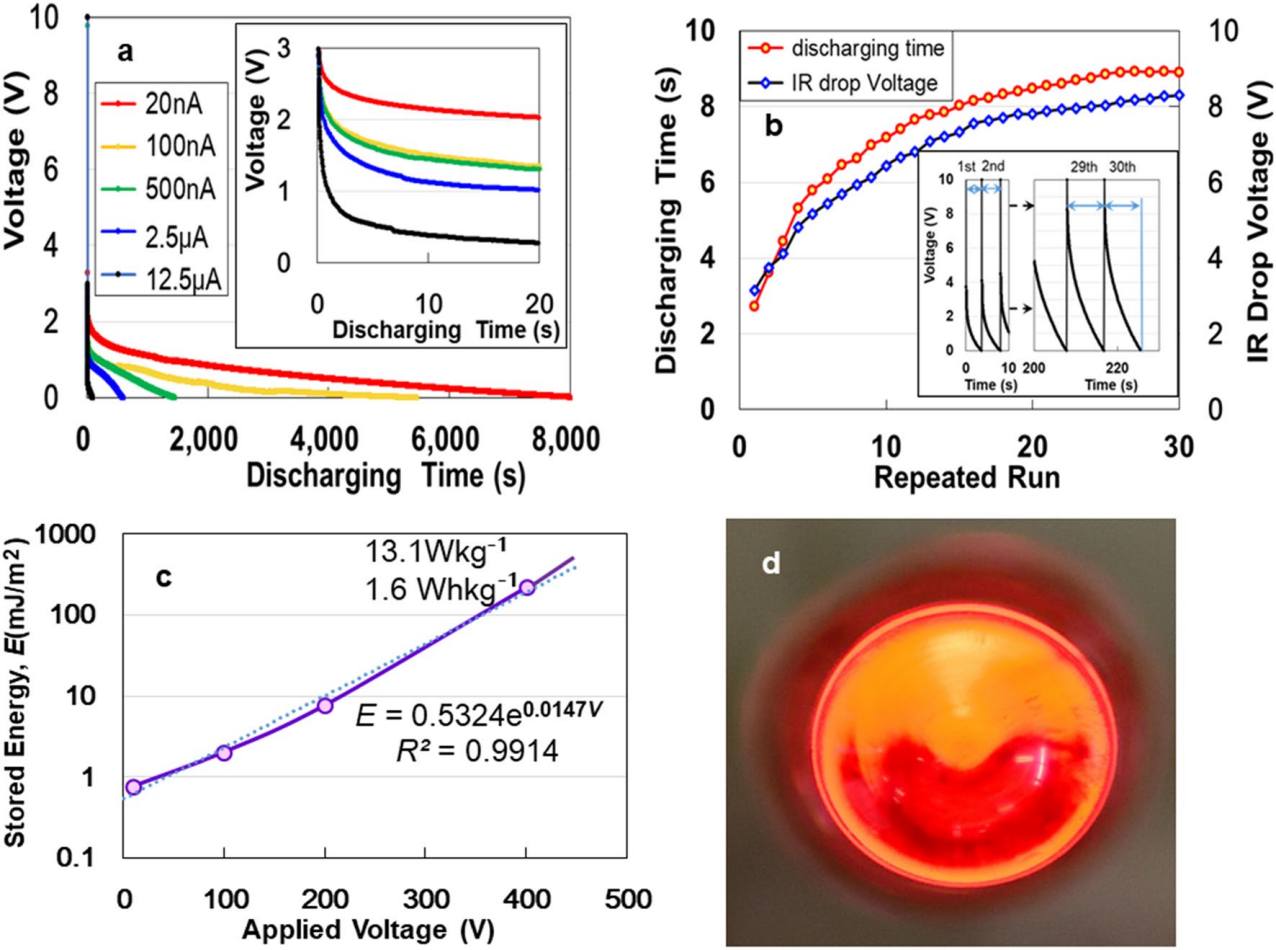
(**a**) Discharging behaviours of the ACF device for constant currents of 20 nA, 100 nA, 500 nA, 2.5 μA, and 12.5 μA after 2 mA–10 V charging for 50 s. (**b**) Discharging time and *IR* drop for 2 mA-rapid charging/1μA-discharging up to 30 times. (**c**) Applied voltage dependency of stored energy. (**d**) An LED powered by the ACF device.

The ACF specimens with a surface area of 2 cm^2^ exhibit discharging behaviours under constant currents of 20 nA–12.5 μA, after 2 mA–10 V charging for 50 s (Fig. 1a). All curves show an ohmic *IR* drop to approximately 3 V through a gradual decrease (see comparison with ATO, APP, and AAO in Supplementary Information [hereafter, referred to as (SI)] Fig. S4). The *IR*- drop is due to internal charging of unsaturated cells as well as the EDLC^18^. We repeated the test under 2 mA-rapid charging/1μA-discharging up to 30 times (Fig. 1b). The discharging time increases and the *IR*-drop decreases with every cycle repeated. The characteristic of increase in electric storage with increasing charge/discharge frequency is similar to that of EDLCs and LIBs which take a long time to charge under constant current^18^. Contrary to conventional charging, we tried to enhance the charge by voltage application for a 10-s duration. The applied voltage (*V*) dependency on the stored electric energy (*E*) (Fig. 1c) can be expressed as *E* = 5 × 10^−5^e^0.014 V^. The maximum energy at 400 V corresponds to 221 mJm^−2^ (13.1 Wkg^−1^, and 1.6 Whkg^−1^). We illuminated a red LED to prove the electric energy storage of ACF. The device with a surface area of 2400 mm^2^, lit the LED for 1 s (Fig. 1d) after it was charged with 2 mA at 10 V. Further gains might be attained by integrating CNF specimens with a nano-electromechanical system (NEMS) (see Fig. S9 in SI).

### Complex evaluation of electric storage and *I–V* characteristics

**Figure 2:**
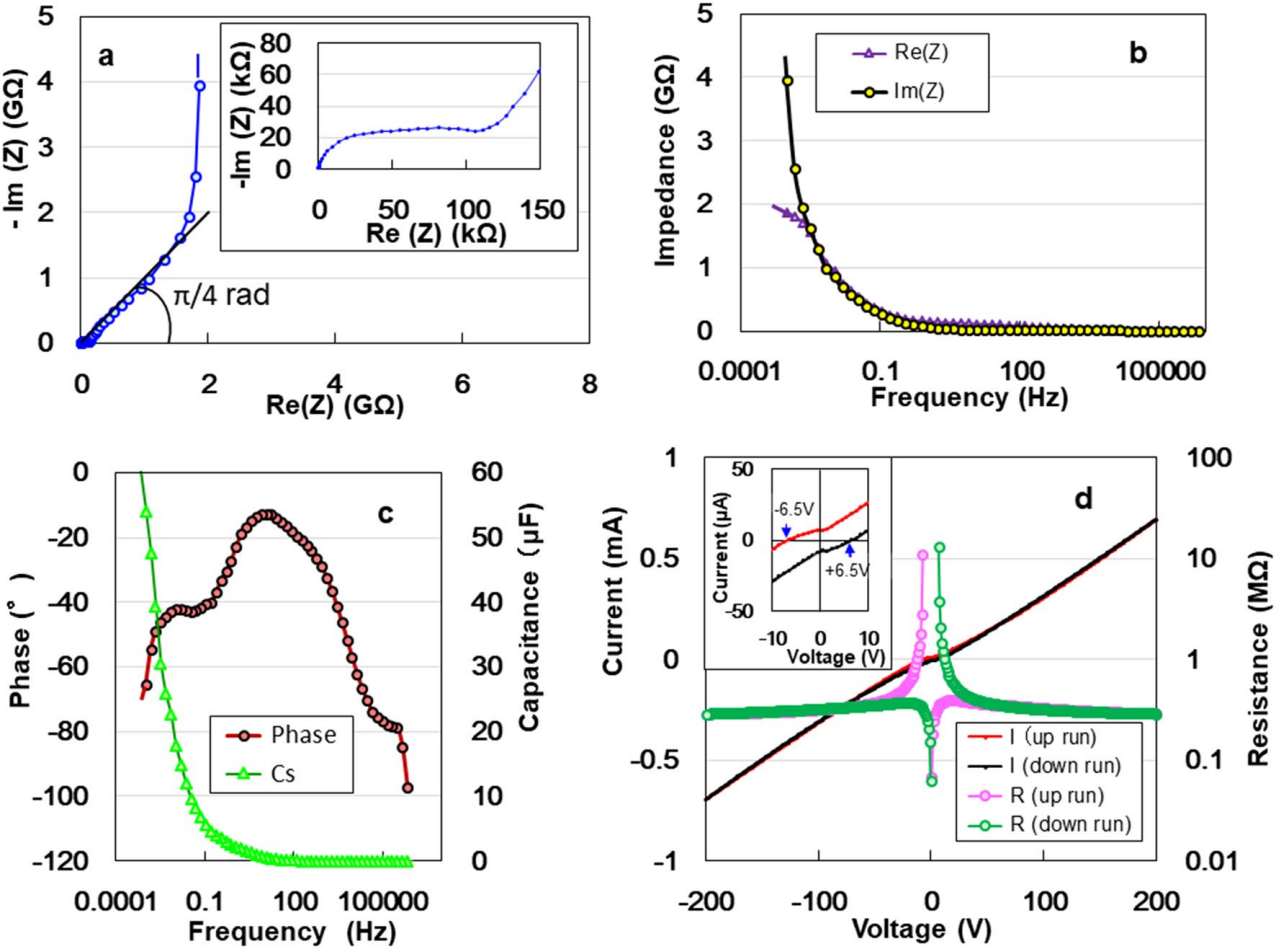
Non-destructive analysis of the electrostatic contribution of the specimen. (**a**) Nyquist plot as a function of frequency for the ACF device. (**b**) Real and imaginary impedances. (**c**) Frequency dependence of phase angle and series capacitance. (**d**) *I–V* and *R–V* characteristics between − 200 and + 200 V.

To analyse non-destructively the electrostatic contribution of the specimen, we measured the AC impedance from 1 to 1 MHz. A Nyquist plot of the impedance data is shown in Fig. 2a. The impedance variation with frequency data of ACF follows the combined pattern of a quadrant, straight horizontal line (inset of Fig. 2a), line with a slope of π/4 rad, and near-vertical line, attributing to a series-*RC* circuit^9,11,12^. The π/4 rad region (Warburg region) is a consequence of the distributed resistance/capacitance in the porous electrode^19^. The imaginary and real impedances rapidly increase up to 4 and 2 MΩ in the lower-frequency region of the Bode diagram, respectively (Fig. 2b). Moreover, the decrement in phase angle to -90°with decreasing frequency is another evidence of DC charging (Fig. 2c). This means that each capacitor on the ACF specimen is connected to a series circuit, C $={\sum }_{k=1}^{n}Ck=nC$. Therefore, the ACF offers an approximately ideal electric distributed constant (EDC) structure for enhancing electrical power storage. The experimental curve of series capacitance *Cs* can be expressed as *Cs* = 1.85f^−0.494^ (r^2^ = 0.9984), where modulating the frequency *f* allows a considerable increase in DC capacitance. Figure 2d represents double *I*–*V* and *R*–*V* characteristics between − 200 and + 200 V in air. The curves are asymmetric relative to zero bias (inset of Fig. 2d), which is similar to the Coulomb blockade behaviour^15^. The current *I* reached zero at − 6.5 and + 6.5 V upon increasing and decreasing the applied voltage *V*, respectively. The zero current at − 6.5 and + 6.5 V correspond to the emission of electrons from the negative to the positive electrode and from the concave to the convex portions, respectively. This proves the electricity switching effect in rechargeable dry solid AAO supercapacitors^12,15,17^.

### Structural morphologies and surface characteristics by TEM and AFM

**Figure 3:**
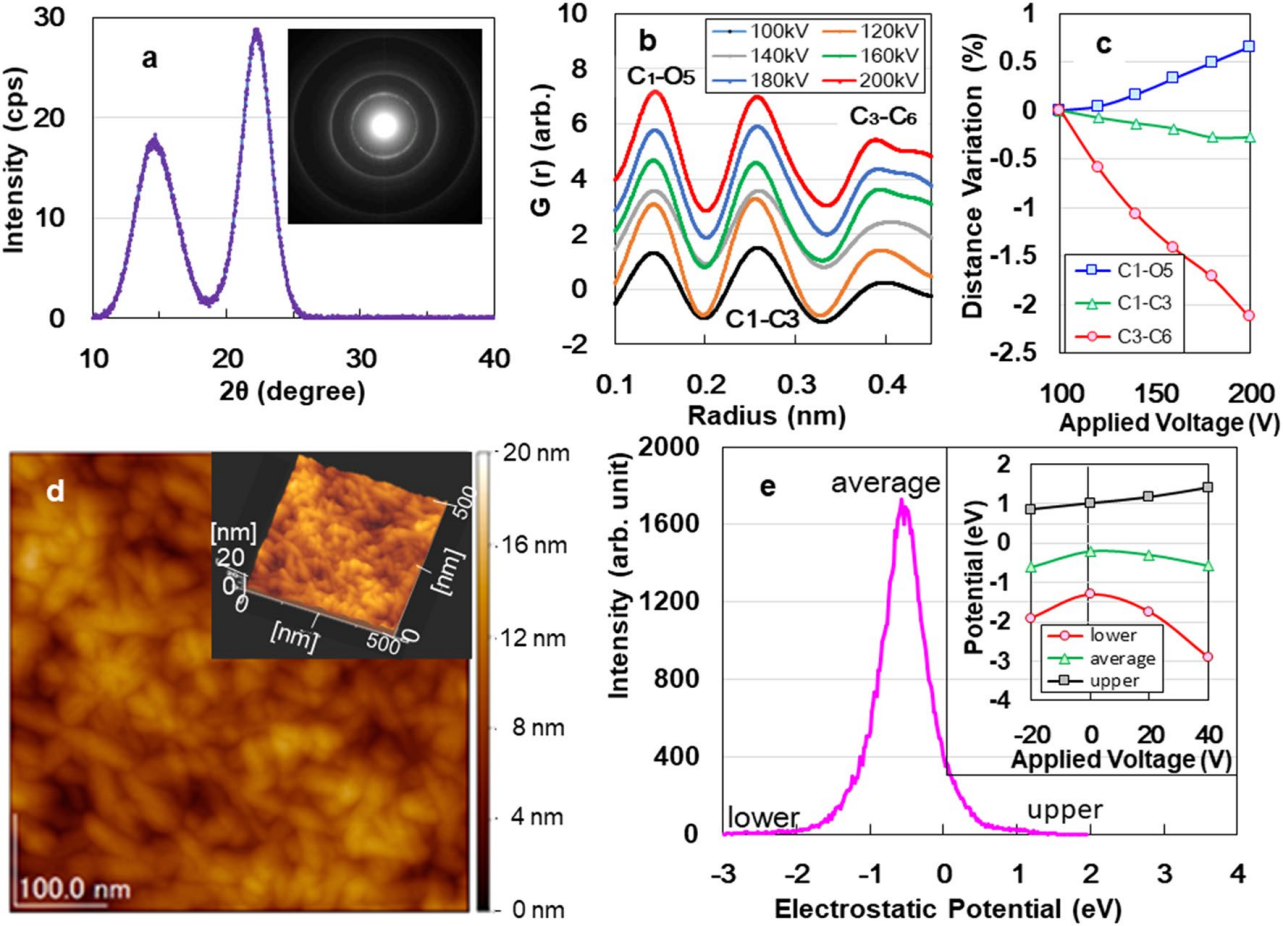
(**a**) XRD analysis of ACF specimen. (**b**) Changes in atomic pair distribution functions (PDFs) under 100–200 kV irradiation at a rate of 3 nA/m^2^. (**c**) Change rates of bonding distance as functions of applied voltage. (**d**) AFM image of the ACF surface. (**e**) Histogram of the potential distribution at + 40 V. Inset in (**a**) SAED pattern, (**d**) three-dimensional AFM image, (**e**) electrostatic potential distributions for ACF surface when the applied voltage changed from − 20 V to + 40 V.

We investigated the structural morphologies and surface characteristics of the ACF specimen. The wide-field X-ray diffraction pattern (Fig. 3a) shows that the specimen consists of an amorphous cellulose phase, characterised by two broad peaks at approximately 16 and 22°. Few nanocrystals are recognised from the continuous Debye rings in the selected-area electron diffraction pattern (inset of Fig. 3a) (corresponding to the red circle zone of Fig. S2 in SI). However, the contribution of nanocrystals to electric energy storage is unclear (SI Fig. S5). Figure 3b shows the changes in atomic pair distribution functions under a strong irradiation of 100–200 keV (SI Fig. S3). The intensity peaks of the C_1_–O_5_, C_1_–C_3_, and C_3_–C_6_ bonds are at approximately 0.14, 0.26, and 0.39 nm, respectively^20^, and the corresponding change rates of distance as functions of the applied voltage are presented in Fig. 3c. Electroadsorption^21^ causes volume shrinkage of C_3_–C_6_ bonds to 2% due to a -1.98 GPa Maxwell compressive stress (electric field stress) (SI S6). This could be caused by the offset effect of positive polar C_6_=O_6_ radicles of carbonyl groups^11,22^, with a rotation of C_6_–sodium carboxylate side chain about the C_5_–C_6_ bond^23^. Amorphous structure prefers to rotate. Figure 3d shows an atomic force microscopy (AFM) image of the surface structure of specimen, with a convex diameter of 17.9 nm and a concave diameter of 13.1 nm. The fibrous appearance of the outer-surface resembles the uneven surface of ATO^9,10^, APP^11^, and AAO^12,13,15^ (SI Fig. S6). When the applied voltage changed from − 20 V to + 40 V in 600 s in air (SI Fig. S7), the electrostatic potential distribution histogram shifted to negative values (Fig. 3e). The negative shift (inset of Fig. 3e) can be attributed to the electron emission from the negative cantilever and an electrostatic induction from the positive cantilever in noncontact AFM.

### Molecular structure and quantum-size effect for electric storage

**Figure 4:**
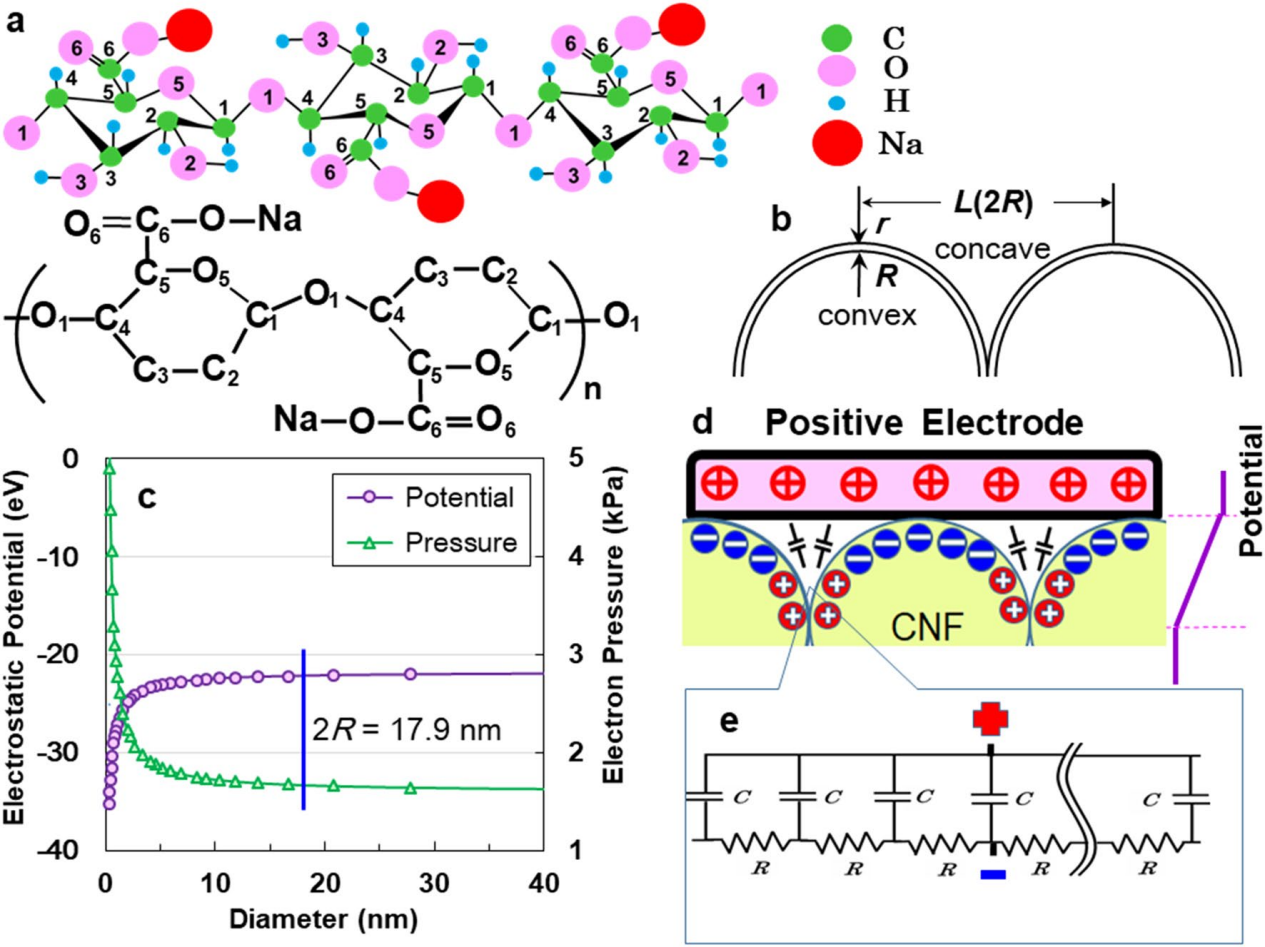
(**a**) Structure of sodium (1→4)-β-D-poly-glucuronate cell with green carbon, pink oxygen, light-blue hydrogen, and red sodium atoms. (**b**) Schematic diagram for calculations based on the Thomas–Fermi statistical method. (**c**) Convex dependences of the electrostatic potential and electron pressure with diameter. (**d**) Schematic representation of the microscopic electric energy storage used in this study. (**e**) The electric distributed constant circuit of the amorphous ACF surface.

The ACF structure, consisting of sodium (1→4)-β-D-poly-glucuronate containing C=O radicles^24^ with permanent dipoles (Fig. 4a)^22,25^, is similar to the APP structure^11^. Therefore, we inferred that the superior electric storage on the uneven ACF specimen is derived from the same quantum-size effect^26,27^ described in Fig. 4b, which induces a relative increase in the combined electrons. This leads to fewer free outer electrons due to the screening effect. Figure 4c presents the convex diameter dependencies of the calculated electrostatic potential and the induced outer electron pressure of carbon atoms surrounding the ACF structure, by help of the Thomas–Fermi screening effect (discussed in the SI S9). The decrease in diameter make increase the negative potential and the positive pressure. The calculated potential is − 22.5 eV at 17.9 nm convex diameter. Therefore, the work function of ACF is negatively higher than those (− 5.5, − 10.1, and − 20.7 eV) of ATO^10^, APP^11^, and AAO^15^, respectively (SI Fig. S9). Figure 4d shows a morphological schematic proposing a possible mechanism for large electrical charges. The uneven surface serves as the EDC circuit (Fig. 4e) with an insulating layer containing tiny capacitors throughout the bulk. Similar to how plant-produced cellulose absorbs atmospheric CO_2_ via photosynthesis, ACF is also expected to capture both positive and negative electricity from the atmosphere, preventing the greenhouse effect.

Thus, the ACF supercapacitor would be suitable for applications of light electricity such as handheld electronic devices, transportation, and renewable energy storage for power grids. However, this paper is the first report that presents the high-performance electric storage of “dry” ACF supercapacitor. We must next investigate electrical characteristics such as specific capacitance, energy efficiency of the charge–discharge process, cyclic stability for practical use.

### Optimised structure of ACF and its electronic role

**Figure 5:**
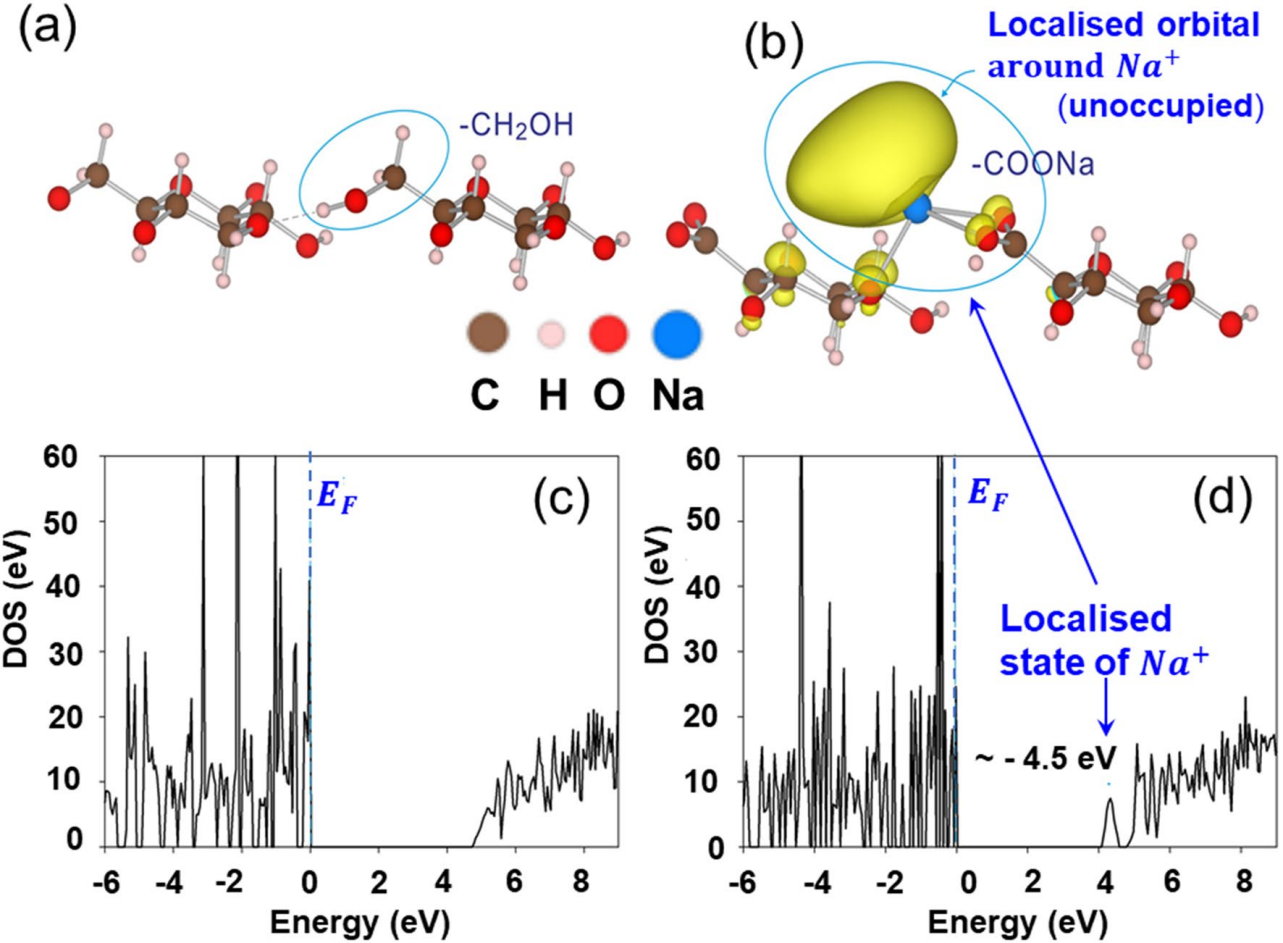
Local structures and density of states (DOS) in C_12_H_20_O_10_ and C_12_H_17_O_11_Na. Isolated electronic state (yellow) locally occurs in vicinity of Na ion.

Finally, we investigate the reason why the CNF possesses superior electric adsorption. We optimized the local structures around CH_2_OH and COONa radicals in TEMPO-oxidised native cellulose (C_12_H_20_O_10_) and NaOH-extracts of TEMPO- oxidised native cellulose (C_12_H_17_O_11_Na), respectively. The local structures of these celluloses are depicted in Fig. 5a,b, respectively. We then simulated the density of sates (DOS) for C_12_H_20_O_10_ and C_12_H_17_O_11_Na, respectively. In sharp contrast to DOS of C_12_H_20_O_10_ with OH radical, an isolated electronic state appears at − 4.5 eV in the band gap for C_12_H_17_O_11_Na with COONa radical (Fig. 5d). This localized state corresponds to the empty 3 s orbital of Na cation shown in Fig. 5b. Here, it should be noted that the localized electrons present near the two-atomic vacancies in the AlO_6_ cluster of AAO induce positive charges (electrostatic effect) on the inside of the insulating oxide surface, resulting in the adsorption of many electrons under electron-beam irradiation^15^. Hence, we infer by analogy that the occurrence of the localized electron in the vicinity of COONa radical induces positive charges (electrostatic effect) on the inside of the insulating ACF surface, leading to high adsorption of many electrons from the atmosphere and in vacuum. Thus, the COONa radical in flexible ACF plays an important role for superior electric adsorption.

## Conclusions

We demonstrated the high-performance electric energy storage (221 mJm^−2^, 13.1 Wkg^−1^) of “dry” amorphous nanocellulose fiber supercapacitor with nanometre-sized cavities and high work function, based on the quantum-size and enhanced electroabsorption effects, respectively. From appearance of localized electrons, C_6_–sodium carboxylate (COONa) radicals in ACF play crucial role (electrostatic effect) for high-performance electric storage. The integration of the film with NEMS is likely to provide potential applications in light electricity such as handheld electronic devices, transportation, and renewable energy storage for power grids.

## Methods

TEMPO-oxidized CNFs (COONa content: 1.48 mmolg^−1^, TC-01A) with 3-nm diameters were prepared by Nippon Paper Industries. The 10-μm-thick ACF specimen was fabricated on the Al substrate via slip casting. Sample structure was examined by X-ray diffraction (XRD) in reflection mode with monochromatic Cu Kα radiation. To avoid destroying of nanofibrils under strong and long-term electron-beam irradiation, we conducted selected-area electron diffraction (SAED) analyses at 100–200 keV with the electron density of 3 nA/m^2^. Electron-refraction and -irradiation were performed using transmission electron microscopy (JEM-2100, JEOL). The noncontact-scanning Kelvin probe-atomic probe microscopy (NC-AFM, JSPM-5200, JEOL), based on the measurement of the electrostatic force gradient was applied to measure the absolute electrical potential between the Pt-coated cantilever tip from − 100 to + 100 V and the ACF surface as the work-function difference. The AC impedance and DC charging/discharging behaviours of each RC combination and illumination test of a red LED light (2 V–100 μA) were analysed using the galvanostatic charge/discharge measured using a potentiostat/galvanostat (SP-150, BioLogic Science Instruments) with DC’s of 10 V, 10 pA ~ 100 mA for ~ 900 s and charging currents of 10 mA at 293 K. A 2 × 10^–4^ W red LED lump was used to verify the electric energy storage. The optimised local atomic configurations of the C_12_H_20_O_10_ and C_12_H_17_O_11_Na were determined through a plane-wave-based first-principles density functional calculation (VASP 5.3)^28^.

## Supplementary Information

Supplementary Information
