# Supplementary material for: Amorphous cellulose nanofiber supercapacitors
Source: Sci Rep. 2021 Mar 19;11:6436. doi: 10.1038/s41598-021-85901-3 (PMC7979786; doi:10.1038/s41598-021-85901-3)
Supplement: Supplementary file 1 — Supplementary Information [file 41598_2021_85901_MOESM1_ESM.docx]

**SUPPLEMENTARY INFORMATION**

**Amorphous Cellulose Nanofiber Supercapacitors**

Mikio Fukuhara^1^, Tomoyuki Kuroda^1^, Fumihiko Hasegawa^1^, Toshiyuki Hashida^2^, Mitsuhiro Takeda^3^, Nobuhisa Fujima^4^, Masahiro Morita ^5^ & Takeshi Nakatani^5^

^1^ New Industry Creation Hatchery Center, Tohoku University, Sendai, Japan, 980-8579

^2^ Fracture and Reliability Research Institute, Graduate School of Engineering, Tohoku University, Sendai, Japan, 980-8579

^3^ National Institute of Technology, Sendai College, Natori, Japan, 981-1239

^4^ Faculty of Engineering, Shizuoka University, Hamamatsu, Japan, 432-8561

^5^ Cellulose Nanofiber Research Laboratory, Nippon Paper Industries, Fuji, Japan, 417-8520

**S1. Methods**

Once-dried bleached hardwood kraft pulp fibers were used as the cellulose source for 2, 2, 6, 6-tetramethylpiperidine-1-oxyl radical (TEMPO)-mediated oxidation at 293 K under aqueous conditions. TEMPO-mediated oxidation and subsequent mechanical distribution were performed according to a previous reported method^29,30^. The resulting TEMPO-oxidized cellulose nanofiber (TOCN), with the H ions of its carboxyl groups replaced by sodium ions (-COONa), had an average diameter of 3 nm (Fig. S1, S2) and carboxyl content of 1.5 m mole/g. The 0.5% (w/v) TOCN/water dispersion was converted to a film with approximately 10 μm thickness on Al sheet with 25 μm. The TOCN films obtained were dried in a ventilated oven at 323 K overnight^30^. The complete picture (30 × 45 mm) of the transparent TOCN sheet is presented at insert of Fig. S1.

Sample structure was examined by X-ray diffraction (XRD) in reflection mode with monochromatic Cu Kα radiation. To avoid destroying of nanofibrils under strong and long-term electron-beam irradiation, we conducted selected-area electron diffraction (SAED) analyses at 100–200 keV with the electron density of 3 nA/m^2^. Observation of topography images and determination of diameter were carried out by a noncontact atomic force microscope (NC-AFM, JSPM-5200, JEOL). The specimens (12 mm wide, 25 µm thick, and 17 mm long) with double–oxidized surface was mechanically sandwiched by two Al electrodes. Current-voltage (*I-V*) and resistivity-voltage (*R-V)* characteristics were measured by DC voltages from -200 to 200 V in air at a sweep rate of 1.24 V/s at room temperature, using a Precision Source Measure Unit (B2911A, Agilent). Storage of charge electricity by constant voltage was carried out for 10 sec at room temperature, using DC Voltage Current Source (6166, ADCMT). The charging/discharging behaviours of each *RC* combination and illumination test of a red LED light (2V-100 μA) were analysed using the galvanostatic charge/discharge measured using a potentiostat/galvanostat (SP-150, BioLogic Science Instruments) with a DC supply of 10 V and charging currents of 10 mA at 293 K. All electrical measurements were carried out in an Al shield box to prevent electromagnetic interference from influencing the results.

Fig. S2 Transmission electron microscope (TEM) image of CNF specimen observed at 200 kV.

Fig. S1 Frequency distribution of CNF

**S2. TEM image of CNF**

TEM image of the CNF specimen observed at 200 kV is presented at Fig. S2. The cellulose structure is characterized by a mix structure of a small quantity (~30%) of irregular lined-up nanofibrils and the most part (~70%) of non-fibrous fibrils. Cellulose bundles are tied up with nanofibrils of 0.3-0.4 nm in diameter. The CNF consisting of cellulose bundles is approximately 3 nm in diameter (see Fig. S1).

**S3. SAED patterns of CNF**

The SAED patterns (Fig.S3 a–f) observed in red circle zone of Fig. S2 depict continuous Debye rings for electron-beam irradiation between 100 and 200 keV, suggesting an existence of nanocrystals phases. SAED patterns for irregular lined-up nanofibrils in red circle zone in Fig. S2 are shown at Fig. S4, indicating partial strong streak in inner most refracted ring. Although Chun *et al*.^8^ reported crystalline-amorphous structures of CNFs comprising closed-packed polysaccharide chains with β-(1➛4)-D-glucopyranose repeat unit, these patterns suggest that the irregular nanofibrils are nanocrystalline phase with small crystallinity^31,32^. From radii of rings, we could calculate face spacing of 0.296, 0.502, and 0.858 nm for the nanocrystals. Judging from TEM image in Fig. S2, the CNF specimen used in this study mainly consists of amorphous cellulose constructed with non- fibrous fibrils. Thus, we termed the CNFs as amorphous cellulose fibers (ACFs).

Fig.S3(a)**—**(f): SAED patterns of CNF specimen under electron-beam irradiation at 100, 120, 140, 160, 180, and 200 kV with the electron density of 3 nA/m^2^.

Fig. S4 TEM images (**a** (160 keV), **c** (200 keV)) and SAED patterns (**b** (160 keV), **d** (200 keV)) for irregular lined-up nanofibrils at 160 and 200 keV.

**S4. Discharging behaviours of ATO, APP, AAO and ACF**

The discharging behaviours of ACF specimens with a surface area of 0.1 cm^2^ under a constant current of 1 nA after the charging of DC currents of 1 mA for 240 s is depicted in Fig. S5, along with those of ATO, APP and AAO. The discharging time (stored energy) of ACF is approximately 1/5, 3000 and 100 times of AAO,^12, 15^ ATO^9, 10^ and APP^11^, respectively. The discharging behaviour of ACF is characterised by large *IR* drop and long discharging time. The potential drop is caused by the current passing through the resistive elements in the equipment circuit of the matrix. However, the large *IR* drop of CNF was improved by repeated charging/discharging runs, as can been seen from Fig. 1b. Furthermore, application of voltage extremely enhanced charging ability of ACF (see Fig. 1c).

Fig. S5 Discharging behaviours of ATO, APP, AAO and ACF at constant current of 1nA after 1 mA-10 V charging for 240 s.

**S5. Discharging times of ACF and CNF specimens**

We compared discharging time for specimen of ACF mainly constructing of amorphous non-fibrous fibrils (Fig. 3a) with that of CNF mainly consisting of irregular lined-up nanofibrils with nanocrystals (inset of Fig. S6). The results are presented at Fig. S6. It is clear that a specimen fabricated from CNFs mainly comprised of nanocrystalline nanofibrils does not reveal superior electric storage compared with amorphous granular CNF one. However, the reason is not clear yet.

Fig. S6 Comparison with discharging time of ACF (Fig. 3) device and CNF (inset) one at constant current of 10 nA after 2 mA-10 V charging for 50 s.

**S6. Compressive force acting on a** **sodium (1➛4)-β-D-poly-glucuronate cell (Fig. 4a) when strong electron beams irradiate on the surface of ACFs**

When the strong irradiation electrons charges on surface of the insulating sodium (1➛4)-β-D-poly-glucuronate cell, a compressive electric field stress (Maxwell stress) is given by the following equation^33^,

P = $\frac{1}{2}ɛE^{2}$, (S1)

*ɛ* = $ɛ_{0}$*ɛ_CNF_* (S2)

where *E*, ɛ and $ɛ_{0}$ are electric field strength for thickness of 25 μm, permittivity and permittivity of free space (8.85 4×10^12^ F/m), respectively. $ɛ_{CNF}$ is permittivity of CNF (7, Ref. 6). From Eqs. (S1) and (S2), we can calculate the compressive stress when applied electron irradiation voltage is 200 keV:

*E* = $\frac{200000}{2.5 x {10}^{-5}}$ = 8 × ${10}^{9}$ Vm^-1^ (S3)

P = — $\frac{1}{2}ɛE^{2}$ = —$\frac{1}{2}{ɛ_{0}ɛ_{Al}E}^{2}$= — $\frac{7\times8.854 \times{10}^{-12}\times{{(8\times10}^{9})}^{2}}{2}$ = —1.983 GPa. (S4)

We reported that AAO indicates a rocking-chair-type charge-breathing effect accompanied by a volume shrinkage of approximately 4% under strong electron-beam irradiation (adsorption) from 100 to 180 keV (Ref. 15). Hence, by analogy, we infer that the ACF has also a ‘rocking -chair-type’ electric storage system similar to a breathing lung.

**S7. Comparison with surface structures of AAO, AAO and APP**

In comparison with surface structure (Fig. 3d) of ACF, Fig. S7 shows a three-dimensional AFM images of the surface structures for AAO^12,14,15^, ATO^9,10^ and APP^11^. The ACF structure is similar to those of AAO, ATP and APP which are characterised by nanometre-sized uneven surfaces.

1. AAO (*d* = 21nm) (b) ATO (*d* = 36 nm) (c) APP (*d* = 28 nm)

Fig. S7 AFM images of surfaces for **a**, amorphous alumina (AAO), **b**, amorphous titania (ATO) and **c**, amorphous perfluorinated polymer (APP).

.

**S8. SKPM equipment for application of voltage**

We investigated the electroadsorption effect of ACF under voltage application of SKPM

in air. When the applied voltage varied from -20 to + 40 V (see Fig. S8 **a**, **b**), we observed electrostatic potential distributions. SKPM images after 600 s for application of -20, 0, +20, and +40 V are presented at Figure S8 **c**, **d**, **e**, and **f**, respectively, showing negative shift of electrostatic potential regardless of negative and positive voltages.

Fig. S8 Schematic views of SKPM equipment for application of a negative 20 V(**a**) and positive 40 V(**b**). **c**, Histogram variation of electrostatic potential for voltage application of -20, 0, +20, and +40 V. SKPM images for voltage application of -20 (**d**), 0 (**e**), +20 (**f**), and +40 V (**g**).

**S9. Calculating the electrostatic potential of C on a nanometre-sized uneven surface using the Thomas-Fermi statistic method**

Our interest lies in studying the electrostatic adsorption of C atoms surrounding the CNF with the nanometre-sized uneven surface with the quantum-size effect in view of the Thomas-Fermi (TF) electronic screening theory. The TF model has been applied to approximate calculations of potential fields and charge densities in elements as a function of lattice spacing. To the best of our knowledge, however, no detailed investigation has been conducted into the quantum-size effect for electric storage.

In general, nanoparticles with a particle size below 100 nm are characterised by a significant paucity in the ratio of chemical bonds in the particle surface. This suggests an increase in free electrons coming from the outer *s* and *p* subshells and resulting in a relative decrease in the inner subshells in nanoparticles. This physical picture explains the lattice expansion of nanoscale compound particles from the neutralization of the screening effect cause by the decreased binding-electron ratio^34^. By reverse analogy, then, we can calculate the electrostatic potential and the electronic pressure of C atoms surrounding the C_5_H_7_O_3_-COONa molecule.

When we assume that the convex portion is almost a half-sphere, the ratio *η* of the topmost atomic layer volume *V*’ to the half-sphere volume *V* increases as the half-sphere diminishes in size:

*η* = *V’/V*= 2*πR^2^r/*(4/6)*πR^3^ =* 3*r/R,* (S5)

where *r* and *R* are radii of the layer atom and the sphere, respectively. Because the outermost bonding of the surface atoms is missing, the missing electrons are apparently free. The ratio of free electrons to binding electrons increases as the size decreases, suggesting a relative decrease in the binding electrons. Because the free electrons squeeze into the inner binding-electron region in nanometre-sized metallic particles^35^, the density *ρ* of the electrons associated with rigid bonding is calculated as follows:

*ρ* ＝ *ρ_0_* (1+*η*), (S6)

where *ρ_0_* is the electron density of the “bulk” atom.

The following relation derives the screening length 1/*λ*^34^:

*λ^2^* = 4*ρ*^1/3^*/a_o_,* (S7)

where *a_o_= h*^2^*/ me*^2^ is the radius of the first Bohr orbit of a hydrogen atom. According to TF approximation, the screened Coulomb potential *φ*(r) is written in the following form:

*φ*(r) = *qe^-λr^*/*r*. (S8)

When the screened Coulomb potential, the screening length, the electron density, and the atomic radius of the “bulk” and nanosphere atoms is *φ_o_*, 1/*λ_o_, ρ_o_, r_o_*, and *φ_1_*, 1/*λ_1_, ρ_1_, r_1_*,

respectively, we can solve

*φ*(*r_o_*) = *φ*(*r_1_*) (S9)

for *r_1_* under an electrostatic equilibrium in potential at *r_o_* and *r_1_*, using the T-F table^36^*φ_0_*(*x*).

We then consider an electronic contribution for the electric storage in terms of the bonding character of C atom, using their electrostatic potential. Because outer electrons in C atom are unsettled in the electronic structure with discrete permitted energies, several free electrons can move around the C atom. According to an ideal gas model, the pressure of the material arises almost entirely from the electrons, both because they outnumber the nuclei and because of their relatively small mass^37^. In electronic kinetic theory, the relation between electronic pressure *P* and the total number of electrons per unit volume *n* is given as follows:

*P* = $\frac{1}{5}({\frac{3}{8\pi})}^{\frac{2}{3}}\frac{h^{2}}{m}n^{\frac{5}{3}}$. (S10)

The number *n* is related to effective potential energy *U* of the outer electrons as follows:

*n* = $\frac{8\pi}{3h^{3}}{(-2mU)}^{\frac{3}{2}}$. (S11)

Eqs. (S10) and (S11) can then be combined as follows:

*P* = $\frac{8\pi}{15h^{3}}m^{\frac{3}{2}}{(-2U)}^{\frac{5}{2}}$, (S12)

where *h* is a Plank constant and *m* is the mass of the electron.

From Eqs. (S9) and (S12), we can calculate the electrostatic potential and the induced outer electronic pressure of the C atom surrounding the CNF. We used the average value 0.1395 nm (Ref. 38) of single and double bond as the ionic radius of C.

**S10. Comparison with convex dependency for ACF, AAO, APP and ATO**

We compare electrostatic potentials of ACF, AAO, APP and ATO as a function of convex diameter on their surfaces. These results are shown in Fig. S9. The work function of ACF is negatively larger than those of AAO, APP and ATO.

Fig. S9　Convex dependences of the electron　potentials　for　the　convex　portion　of　CNF　in　comparison　with　the　convex　dependences　of ATO, APP and AAO.

**S11　Fabrication of three-dimensional device by nano-electro mechanical system (NEMS)**

One enhancement way for electric storage ability is device fabrication by NEMS presented in Fig. S10.

Fig. S10 Device fabrication process by NEMS method.

**References**

^29.^ Saito, T., Kimura, S., Nishiyama, Y. & Isogai, A. Cellulose nanofibers prepared by TEMPO-mediated oxidation of native cellulose, *Biomacromolecules* **8**, 2485‒2491 (2007).

^30^ Fukuzumi, H., Saito, T., Iwata, T., Kumamoto, Y. & Isogai, A., Transparent and High Gas Barrier Films of Cellulose Nanofibers Prepared by TEMPO-Mediated Oxidation, *Biomacromolecules* **10**, 162‒165 (2009).

^31^ V. Petkov, Y. Ren, S. Kabekkodu, D. Murphy, Atomic pair distribution functions analysis of disordered low-*Z* materials, *Phys. Chem. Chem. Phys*. **15**, 8544‒8554 (2013).

^32^ A. D. French and M. S. Cintrón, Cellulose polymorphy, crystallite size, and the Segal crystallinity index*, Cellulose*, **20**, 583‒588 (2013).

^33^ Sadd, M.H. Displacement Potentials and Stress Functions in Elasticity (Elsevier, New York, 2005), p. 347‒369.

^34^ Kittel, C. *Introduction to Solid State Physics*, 4th edition (John Wiley & Sons, New York, 1971), p.279.

^35^ Fukuhara, M. Lattice expansion of nanoscale compound particles. *Phys. Lett. A.* **313**, 427‒430 (2003).

^36^ Condon, E. U. & Odabaşi, H. *Atomic Structure* (Cambridge University Press, London, 1980), p.454.

^37^ Hamann, S. H. *Physico-Chemical Effects of Pressure* (Butter Worths Scientific, London, 1957), p. 59.

^38^ International Tables for X-ray Crystallography, International Union of Crystallography (Kynoch Press, Birmingham, England, 1968), p. 276.
